# Differences between doctors of medicine and dental medicine in the perception of professionalism on social networking sites: the development of the e-professionalism assessment compatibility index (ePACI)

**DOI:** 10.1186/s12910-022-00870-0

**Published:** 2022-12-06

**Authors:** J. Viskić, M. Marelić, L. Machala Poplašen, T. Vukušić Rukavina

**Affiliations:** 1 Department of Fixed Prosthodontics, School of Dental Medicine, University of Zagreb, Zagreb, Croatia; 2 Andrija Štampar School of Public Health, School of Medicine, University of Zagreb, Zagreb, Croatia

**Keywords:** Social networking sites, e-Professionalism, Medicine, Dental medicine, Medical doctors, Dental medicine doctors

## Abstract

**Background:**

Social networking sites (SNSs) have penetrated all aspects of health care professionals’ (HCPs’) professional and private lives. A new term, e-professionalism, has emerged, which describes the linking of traditional values with this new dynamic online environment for HCPs. The four aims of this study were: (1) to examine their SNS prevalence and usage habits, (2) to examine their perception of e-professionalism, (3) to develop an e-professionalism assessment compatibility index and (4) to investigate their tendencies and differences in values of the e-professionalism assessment compatibility index (ePACI).

**Methods:**

A cross-sectional study was conducted among MDs and DMDs in Croatia via email using a questionnaire. The questionnaire was distributed to all MDs and DMDs who were members of their respective chambers. In addition to descriptive statistics, Chi-square or Fisher’s exact test when appropriate, t-test, ANOVA and Mann–Whitney U tests were used to determine differences between groups. A principal component analysis (PCA) with varimax rotation was used to investigate dimensionality. Results of the PCA were compared to the coding based on the Social Media e-Professionalism rubric in order to create the ePACI.

**Results:**

Of the 1013 gathered responses, 753 were from valid SNS users and suitable for further analysis. Facebook (91.6%) and Instagram (63.1%) were the predominant SNSs used. Both groups have a good understanding of e-professionalism. The newly developed ePACI deviates slightly in the “conservative” direction in the cases of both, MDs (t_506_ = 19.033, *p* < 0.001) and DMDs (t_245_ = 12.991, *p* < 0.001). HCPs who are older (r = 0.308, *p* < 0.001), who have fewer SNS profiles (r_s_ = −0.142, *p* < 0.001), and who access their profiles less frequently (r = −0.166, *p* < 0.001) have statistically significantly more conservative ePACI values.

**Conclusions:**

MDs and DMDs in Croatia are frequent SNS users, with Facebook and Instagram being the main SNSs used. Both groups have a good understanding of e-professionalism. The newly developed ePACI deviates slightly towards the conservative side regarding perception of the e-professionalism content for both groups. The development of the ePACI, and its subsequent usage in further research, will have a direct influence in its validation, gathering comparable data, and be able to direct efforts in oversight, regulation and education.

**Supplementary Information:**

The online version contains supplementary material available at 10.1186/s12910-022-00870-0.

## Background

Social media (SM) has a huge impact on our daily lives. It has revolutionized the way we communicate, gather information and socialize. Currently half of the Earth’s population is present on SM with projected numbers for 2025 rising to 4.41 billion users [1]. The millennials are digital natives, having been immersed in online life and SM from an early age [2]. They see it as an extension of themselves, and are even more open to communication via SM than in real life [3]. There is a difference between SM and social networking sites (SNSs). The most widely accepted definition of SM is “a group of online applications that allow for the creation and exchange of content generated by users” [4]. SNSs by definition are “applications that enable users to connect by creating personal information profiles, inviting friends and colleagues to have access to those profiles, and sending e-mails and instant messages between each other” [5]. SNSs include Facebook, Instagram and LinkedIn, and the term is narrower and more precise for what is referred to when considering these online services.

Traditional concepts of professionalism in medicine require healthcare professionals (HCPs) to adopt the identity of medical professional, and enact it at the individual, interpersonal, and societal levels [6, 7]. E-professionalism is a term first used by Cain et al., defined as “attitudes and behaviours (some of which may occur in private settings) reflecting traditional professionalism paradigms that are manifested through digital media” [8]. It is a term that refers to the professional behaviour of HCPs when using digital communication and SM platforms. Guraya et al., in a recent study, argued that an adequate framework for e-professionalism on a “virtue-based”, “behaviour-based” and “professional identity formation-based” framework did not exist [9]. They subsequently developed the medical education e-professionalism framework to address this issue.

In the early stages of SNS development, researchers expressed much reserve and caution regarding these new emerging platforms, suggesting HCPs exercise stricter oversight and caution when using SNSs [10, 11]. Currently, many HCP students are classified as either “millennials” or “Gen Z”, and there has been extensive research into their habits and attitudes toward SNSs [12]. Students of medicine and dental medicine show a high level of understanding of SNS use and e-professionalism [13, 14]. However, there has been little insight into the population of practicing HCPs, who willingly or out of necessity—are increasingly engaging with SNSs. There is also an exceptionally large knowledge gap in the understanding of the similarities and differences, concerning e-professionalism, between MDs and DMDs [15]. These professionally similar yet perhaps motivationally and practically diverging health care professions are adopting the benefits and responding to the dangers of SNSs in different ways [4, 16]. Dentistry is a much more visually driven profession, with aesthetics having become a major motivating force in recent times. This is fuelled by changes in modern society's perceptions of beauty standards. Healthy, white, perfectly shaped and aligned teeth are becoming a social status symbol, with SNSs being the perfect platform to advertise such standard, for both dentists and patients [17–19]. In the field of medicine, cosmetic medicine is also under a similar influence, but it is a proportionately smaller division of medicine [20]. Also, cosmetic medicine may involve private parts of the human anatomy more than dental medicine, so visual representations of procedures, and “before and after” images are still not so widespread. Dentists learned early on that SNSs could be used to educate patients and the general public, and that their habits could also be influenced through SNSs [21, 22]. As the number of SNS users increase potentially more patients can be reached via SNS campaigns promoting dental health practices rather than simply in-office communication. Also, in Croatia DMDs are, on average, more involved in private practice than MDs [23]. The business imperative to maintain contact with existing and future patients can encourage a more open attitude towards new and emerging tools for such improvements. This also opens the possibility for a more financially motivated approach to practising than medical [24, 25].

Results from research show students of dental medicine are less sensitive in response to visual representations of patients on SNSs, even if they reveal patient information, than medical students [13]. Little insight exists into whether this trend continues after graduation and during subsequent clinical practice, where professional boundaries become more apparent and crossing them brings consequences.

A very important factor in examining e-professionalism is the patient. Studies have shown that patients are educating themselves about their health online and using SNSs to investigate their disease, their therapy and also their healthcare provider [26, 27]. Interaction between HCPs and patients is inevitably happening on SNSs, with professional boundaries being blurred and potentially crossed [28].

The benefits of SNSs are emerging rapidly, but so are their dangers [15]. In extreme cases, disciplinary or legal action can ensue with long term consequences [29, 30]. As the digital footprint is permanent, personal or professional posts on an SNS can have an impact on an HCP’s career, both positive and negative [31]. This is why a clear understanding of e-professionalism is necessary. In a recent study, Mosalanejad et al. investigated the concepts of e‑professionalism in medical sciences, and set examples of negative and positive components of the virtual environment and SNSs’ use for HCPs, and also provided suggestions on how to improve them [32–34]. They emphasized the role of education on professional identity and also the need for increasing the cultural awareness of people, and role model presentation [32].

In a study by White et al., a set of questions to explore the perception of online professionalism was developed, and the results demonstrated that students had a developed sense of e-professionalism [12]. These questions were also used by Viskić et al., who produced similar results [13]. However, there can be a difference between the perception of online professionalism and the actual content the HCPs post online. Content analysis of SNS profiles can give a clearer insight into this divergence among HCPs. Several studies have addressed this issue with sometimes controversial results [35–37]. In a retracted paper by Hardouin et al., posts depicting bikinis were considered “potentially unprofessional”, sparking outrage among the HCPs SNS community and resulting in the “medbikini affair” [38, 39]. In the wake of the “medbikini affair” re-examination of the current e-professionalism standards for posting textual, photographic or video content on SNSs became necessary. A novel “Social Media e-Professionalism” (SMePROF) coding scheme, that includes the SMePROF rubric for assessment of unprofessional content on Facebook, has been developed by Vukušić Rukavina et al. [40]. The SMePROF rubric, whose design is based on current perceptions of professional online behaviour, can be used to assess online content and classify it into one of three distinct categories: professional, potentially unprofessional, and unprofessional. As the SMePROF rubric for assessment of unprofessional behaviour can now be perceived as a norm or expected value for e-professionalism, tendencies in any deviation from that norm can be explored: either to more lenient and relaxed perception (liberal), or a more cautious and rigid (conservative) perception of e-professionalism. The importance of this study stems precisely from the gap in knowledge that remains after existing research has investigated what SNS content is unprofessional and how accurately respondents recognize it. Insight into the tendencies and differences among MDs and DMDs regarding liberal or conservative views of e-professionalism has not, to our knowledge, been described in the recent literature. To understand how MDs and DMDs deviate from the norm means to understand how they make mistakes, which is particularly relevant information for possible educational interventions.

Educational efforts on the correct use of SNSs are crucial at all levels of HCP education. This is especially true for the generation of practising HCPs who are not digital natives and could perhaps have difficulty with even the basics of SNS use. As part of a larger long-term research project funded by the Croatian Science Foundation, UIP-05-2017 “Dangers and benefits of social networks: E-Professionalism of health care professionals—SMePROF” [41], this study was envisioned to bridge the knowledge gap that exists in the literature on the prevalence, usage habits, and perception of e-professionalism on SNSs among MDs and DMDs. Also, in this study, we set out to provide new outlooks on e-professionalism and new tools in dealing with e-professionalism problems among MDs and DMDs on SNSs.

## Aim

This study had four distinct aims:

1. To examine the SNS prevalence and usage habits of MDs and DMDs in Croatia.
2. To examine the perception of e-professionalism of MDs and DMDs in Croatia.
3. To develop an e-professionalism assessment compatibility index of MDs and DMDs in Croatia.
4. To investigate tendencies and differences among MDs and DMDs in Croatia regarding values demonstrated by the e-professionalism assessment compatibility index.

## Methods

### Design

A quantitative cross-sectional study on the use of SNSs and attitudes towards e-professionalism among MDs and DMDs was conducted in collaboration with the Croatian Medical Chamber (CMC) [42] and the Croatian Chamber of Dental Medicine (CCDM) [43].

### Study questionnaire

Data were collected by using a survey-specific questionnaire named “SMePROF Project Survey Questionnaire on Social Media Usage, Attitudes, Ethical Values and E-professional Behaviour of Doctors of Medicine and Doctors of Dental Medicine”. The complete study questionnaire is available as Additional file 1.

The questionnaire used in this study was derived from previous studies within the project “Dangers and benefits of social networks: E-professionalism of healthcare professionals—SMePROF [13, 41, 44] where the study population was students of medicine and dental medicine. The questionnaire used in this study was composed of eight instruments that measured (1) sociodemographic characteristics and habits of SNS usage; (2) knowledge of SNSs; (3) reasons for SNS usage; (4) impression management on SNSs; (5) security on SNSs; (6) attitude toward professionalism; (7) attitude toward e-professionalism and (8) perception of e-professional content on SNSs.

The instrument used to measure the perception of e-professional content on SNSs was first developed by White et al. [12]. Using a mixed-method approach, they conducted interviews with students from various healthcare professions (nursing, medicine, dentistry, pharmacy and physical therapy) and constructed, on the basis of identified domains, an instrument of 19 items to measure the perception of unprofessional content on Facebook. This instrument was translated into Croatian, tested on medical and dental students in Croatia during the 2018/2019 academic year and proved to be a valuable tool for understanding perceptions of e-professionalism among this group [13]. In order for this instrument that measures perception of e-professional content to be used with MDs and DMDs instead of students, it underwent validation and minimal modifications by the exclusion of two items that were specific to a student population but were not applicable to the population of MDs and DMDs [44]. After modification the instrument used in this research has 17 items.

The final version of the questionnaire was entered into the online survey-generating application Microsoft Forms. The questionnaire began with informed consent (with an “opt out” option) followed by the questions. Both the study and the questionnaire were approved by the ethical boards of the University of Zagreb School of Medicine (641–01/18–02/01) and the University of Zagreb School of Dental Medicine (05-PA-24-2/2018), these being the institutions in charge of the study. Also, formal approval was obtained from the governing bodies of both the CMC (900-06/20-01/11) and CCDM (900-01/21-01/02) for use of the full mailing lists of MDs and DMDs who were members of the CMC and CCDM.

### Data collection and analysis

The planned sample size was 200 MDs and 200 DMDs, corresponding to a 10:1 ratio between number of observations and the number of variables that were used in the largest instrument in the questionnaire. The planned sample was defined according to a conservative estimate often used for multivariate analyses [45]. Type of sample was a non-probabilistic convenience sample. Participating MDs and DMDs were recruited through two rounds of survey email invitations distributing the questionnaire, which was entered in Microsoft Forms, between 16 February and 13 July 2021. The mailing lists used to distribute the survey link were the official full membership mailing lists of the CMC and CCDM. At the time of the survey, the CMC’s mailing list contained 15,562 email addresses of MDs, and the CCDM’s email list contained 7,616 email addresses of DMDs. The first invitation was sent on 16 February 2021, and a second invitation (reminder) was sent on 4 May 2021. The email included a brief text about the objective of the study, the average response time, and the person and university responsible for conducting the study. Participation in the survey was voluntary; there was no form of incentive to complete the survey. To ensure anonymity, no identification data were collected. By default, data were not collected from Microsoft Forms if the questionnaire had not been completed. Duplicate data were excluded. Subjects who indicated they were not SNS users were redirected to the end of the questionnaire, and their negative response was recorded and not included in further analysis. For the purpose of achieving the four aims of this study, we have analysed data gathered from two instruments of the questionnaire: the first instrument, which measured sociodemographic characteristics and habits of SNS usage, and the eighth, which measured perception of e-professional content.

Demographic data were analysed using descriptive statistics. Comparisons of MDs’ and DMDs’ responses were calculated using the Chi-square or Fisher’s exact test as appropriate for categoric variables, a t-test and the One-Way ANOVA for continuous variables (with Tamhane T2 post-hoc test) as well as the Mann–Whitney U test for ordinal variables. Yates's correction for continuity was employed in Chi-square tests conducted on 2 × 2 contingency tables [46]. Correlations were explored using Pearson’s or Spearman’s correlation coefficients.

### Development of the ePACI index

To understand the underlying dimensions of the instrument to measure perception of e-professional content a principal component analysis (PCA) was conducted. The results were used to get a better understanding of latent components: which items should be considered unprofessional (regardless of perception). A PCA with varimax rotation was used to investigate dimensionality. A two-tailed alpha of 0.05 was used to denote statistical significance. Statistical analysis was conducted in IBM SPSS STATISTICS 25.0.

In order to investigate how MDs’ and DMDs’ perceptions of unprofessional content differ, we have designed a measure called the e-professionalism assessment compatibility index (ePACI). First, we had to devise a scientifically and theoretically sound way to define “the correct answer”, or “the norm”. In other words, we wanted to see whether content that respondents perceived as “unprofessional” really was “unprofessional”, and to what extent they were able to distinguish “professional” content. The correct answer was defined using the SMePROF rubric for the assessment of unprofessional content on Facebook developed in Vukušić Rukavina et al. (SMePROF rubric) [40].

Items in the instrument that measured perception of e-professional content were coded according to the same SMePROF rubric by two independent experts (TVR and JV), and after individual coding the discrepancies were addressed and a consensus was achieved. The items were sorted into three categories: five items were coded as professional, two as potentially unprofessional and ten as unprofessional (Table 1).

**Table 1:** Coding of the instrument “perception of e-professional content” items according to the SMePROF rubric

Items of the instrument perception of e-professional content	Coding according to the SMePROF rubric
A picture of an individual having one alcoholic beverage	PC
Pictures of an individual clearly behaving drunkenly	UPC
Status updates describing substantial alcohol consumption at a party	UPC
Posts depicting illicit drug consumption	UPC
Posts disclosing information about a patient/client	UPC
Photos of a patient/client	UPC
Posts describing an interaction with a patient/client, while not revealing any identifying information	PUPC
Swearing or foul language	UPC
Obscene gestures in photos (the middle finger, etc.)	UPC
Petty criminal activity	UPC
Endorsements of a pharmaceutical or health product without a conflict-of-interest disclosure	PUPC
Posts involving overt sexual content	UPC
Posts containing partial nudity	PC
Displaying your current relationship status	PC
Displaying membership in online groups dealing with controversial issues	PC
Making opinionated comments about controversial issues	PC
Attitudes of superiority or condescending behaviour (assumed because of professional status)	UPC

*PC* professional content, *PUPC* potentially unprofessional content, *UPC* unprofessional content

Potentially unprofessional items, although theoretically and thematically very interesting, were not useful in this analysis and were removed from the ePACI. Therefore, the index was formed using five “professional” and ten “unprofessional” items. ePACI was designed to measure not only perception in line with the expected answer, but also the direction of deviation which is, according to this study’s fourth aim, the investigation of tendencies and differences among MDs and DMDs regarding values demonstrated by the ePACI index. This was achieved by creating an additive index where the correct answer regarding an item (or perception that matches the criteria based on Vukušić Rukavina et al. [40]) was coded as 0, deviating toward professional items was coded as +2, and deviating towards unprofessional items was coded as −1. The fact that professional items were awarded more points is a result of weighting because of the difference in the number of items measuring each type of post (10 unprofessional against 5 professional). This meant that if the respondent answered correctly to all items his/her score would be value marked zero—0. The minimum value of the index is −10 and it could be achieved by missing only unprofessional items, and the maximum value of the index is +10 and it was possible if the respondent missed only professional items. In this paper, with the minimum value of the unstandardized index −10, and the maximal theoretical value of the unstandardized index +10, that would come to:

$${\mathrm{ePACI}}=\frac{i+10}{10}-1$$

Although such extremes are not expected, a deviation from the correct answer can be seen in the index score of each respondent. In order to make the index more flexible and easier to replicate by other researchers it was mathematically standardized to values between −1 and +1, where negative values reflect a deviation in a “liberal” direction (when unprofessional behaviour is considered acceptable), and positive values reflect a deviation in a “conservative” direction (when someone considers acceptable behaviour as unprofessional) using this formula:

$${\mathrm{ePACI}}=2*\frac{i-{{\min}}_{i}}{{{\max}}_{i}-{{\min}}_{i}}-1$$

where ePACI—new index, *i*—unstandardized index, min_i_—minimal theoretical value of the unstandardized index, max_i_—maximal theoretical value of the unstandardized index.

**Fig. 1:**
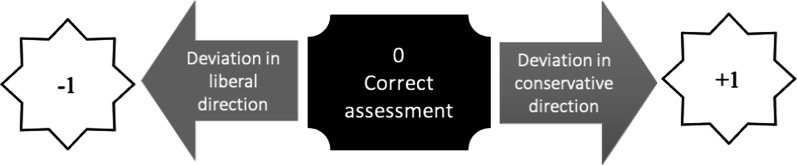
Representation of logic deviation from −1 (liberal) to +1 (conservative) in ePACI values

This logic is illustrated in Fig. 1.

It is important to note that, in this research, the labels liberal/conservative have no intrinsic positive or negative connotations. In this research the deviation of values in either direction represents a deviation from the value-correct perception (value marked zero—0) of e-professionalism.

The correlation of ePACI values with variables of our studied sample was also performed. Gender, type of employment, age, number and type of SNS profiles, and frequency of access were variables compared with ePACI values. Differences were examined between the two groups within the sample (MDs and DMDs) and as a whole. To identify differences related to the age of the subjects, we have divided our sample into four groups. The first group (< 35 yrs. old) falls into the group of digital natives [2]: those who have been exposed to, or who have potentially had access to, SNSs their entire lives. The second group (35–45 yrs. old) are individuals who potentially had access to SNSs early in their careers, possibly right after graduation, and are potentially very well versed in all aspects of online life, but have lived in a world without SNSs. The third group (45–55 yrs. old) are digital immigrants [2], HCPs whose careers have long been based on traditional professional standards, but who are possibly willing to invest time and effort to explore and immerse themselves in SNSs. Finally, there is a group of HCPs (55+ yrs. old) who are in the more mature phase of their careers, have decades of knowledge and experience with professional standards, and may be less than knowledgeable and even potentially fearful of new technologies, or could have a low digital literacy level [15, 47, 48].

## Results

A total of 1013 questionnaires were collected. The response rate was 4.4% (1013/23178). Of the total number of questionnaires collected, 14 subjects’ questionnaires were excluded (nine respondents did not accept informed consent; one respondent reported being 18 years old, which is not consistent with the possible graduation age of MDs or DMDs; four cases were duplicates with identical responses to 45 variables during statistical analysis). Of the remaining 999 questionnaire respondents, 246 (24.6%) reported not using SNSs. Their demographic data and initial responses were recorded but they were not included in further analysis. The remaining 753 completed questionnaires were analysed; 507 (67.3%) of the respondents were MDs and 246 (32.7%) were DMDs. The sample was predominantly female (72.1%), with a median age of 35. Significant differences in demographics between MDs and DMDs were observed. There was a difference in age, where MDs were older than DMDs with an average age of 39.26 as opposed to 36.58 years, respectively (t_642.643_ = 3.552, *p* < 0.001), and in type of employment, with more than two-thirds of DMDs (69.1%) being employed in the private sector compared to only 20.6% of MDs (χ^2^_1_ = 164.481, *p* < 0.001). More complete demographical data are shown in Table 2.

**Table 2:** Distribution of doctors by sex, age and profession

	All doctors N (%)	Medical N (%)	Dental N (%)	Statistical test
Sex				
Male	195 (25.9)	124 (24.5)	71 (28.9)	
Female	558 (74.1)	383 (75.5)	175 (71.1)	1.674, 1, 0.228^[^^1]^
Total	753 (100)	507	246	
Age				
Average	38.39	39.26	36.58	
Median	35	35	35	
Min	24	24	24	3.522, 642.643, 0.000*^[^^2]^
Max	73	73	60	
SD	10.990	11.890	8.598	
Type of employment				
Public sector	475 (63.6)	400 (79.4)	75 (30.9)	164.481, 1, 0.000*^[^^1]^
Private sector	272 (36.4)	104 (20.6)	168 (69.1)	

*p < 0.01

^[1]^χ^2^-test with Yates’ correction

^[2]^t-test

There were 286 (38%) graduate MDs and DMDs, 225 (29.9%) residents and 242 (32.1%) specialists.

The results on SNS use, habits and device preferences are shown in Table 3. There are statistically significant differences in usage between MDs and DMDs on all SNSs studied. DMDs are generally more active on SNSs than MDs, and access SNS sites statistically significantly more often per day (U = 54,641.5, *p* = 0.003). However, both groups (n = 588, 78%) use SNSs intensively at least once per day.

**Table 3:** SNS usage, habits and device preference

	All doctors N (%)	Medical N (%)	Dental N (%)	Statistical test
SNS platforms				
Facebook	695 (91.6)	457 (90.1)	238 (96.7)	9.271, 1, 0.00**^[1]^
Instagram	475(63.1)	292 (57.6)	183 (74.4)	19.350, 1, 0.00**^[1]^
LinkedIn	392 (52.1)	281 (55.4)	111 (45.1)	6.637, 1, 0.01*^[1]^
YouTube	248 (32.9)	183 (36.1)	65 (26.4)	6.584, 1, 0.01*^[1]^
Twitter	78 (10.4)	58 (11.4)	20 (8.1)	1.614, 1, 0.20^[1]^
Snapchat	45(6)	23 (4.5)	22 (8.9)	4.967, 1, 0.026*^[1]^
TikTok	35(4.6)	15 (3.0)	20 (8.1)	8.862, 1, 0.00**^[1]^
Active/passive usage				
Exclusively passive	132 (17.5)	106 (20.9)	26 (10.6)	54,329.5, 0.001**^[2]^
More passive than active	450 (59.8)	297 (58.6)	153 (62.2)	
Half-and-half	115 (15.3)	65 (12.8)	50 (20.3)	
More active than passive	51 (6.8)	36 (7.1)	15 (6.1)	
Exclusively active	5 (0.6)	3 (0.6)	2 (0.8)	
Access frequency				
More than 10 × a day	85 (11.3)	58 (11.4)	27 (11.0)	54,641.5, 0.003**^[2]^
5 to 10 × a day	379 (50.3)	231 (45.6)	148 (60.2)	
Once a day	124 (16.5)	93 (18.3)	31 (12.6)	
2 to 3 × a week or less	165 (21.9)	125 (24.7)	40 (16.3)	
Device				
Smartphone/tablet	699 (92.8)	459 (90.5)	240 (97.6)	11.258, 1, 0.001**^[1]^
Personal computer	54 (7.2)	48 (9.5)	6 (2.4)	

*p < 0.05; **p < 0.01

^[1]^χ^2^-test

^[2]^Mann Whitney U test

The predominant (92.8%) access medium is a mobile device (smartphone or tablet), with DMDs being statistically significantly even more likely to use those devices (97.6% DMDs vs. 90.5% MDs, χ^2^_1_ = 11.258, *p* = 0.001).

Table 4 describes the comparison of perceptions of e-professional behaviour between MDs and DMDs. HIPAA violations (99.1%), criminal activity (98%) and illicit drug use (97.1%) ranked top of the list of posts perceived as unprofessional for both groups. Interesting statistically significant differences between groups are revealed in “Photos of a patient/client” with DMDs being significantly less likely to perceive such content as unprofessional (97.2% MD vs. 86.2% DMD, χ^2^_1_ = 32.119, *p* < 0.001). Similarly, MDs are statistically significantly more likely to perceive “Posts describing an interaction with a patient/client that does not reveal any identifying information” as unprofessional than DMDs (60% MD vs. 40.2% DMD, χ^2^_1_ = 25.097, *p* < 0.001). MDs are also more likely to have negative perceptions of “Endorsements of a pharmaceutical or health product without a conflict-of-interest disclosure” (75% MD vs. 58.9% DMD, χ^2^_1_ = 19.354, *p* < 0.001), and to “A picture of an individual having one alcoholic beverage” (35.7% MD vs. 22.8% DMD, χ^2^_1_ = 12.258, *p* < 0.001) as unprofessional.

**Table 4:** Comparison of e-professional behaviour perception between MDs and DMDs

Online behaviour	Classifying as "unprofessional"			
	All doctors N (%)	MD N (%)	DMD N (%)	χ^2^, df, *p*
Posts disclosing information about a patient/client	746 (99.1)	504 (99.4)	242 (98.4)	0.965, 1, 0.326
Petty criminal activity	738 (98)	496 (97.8)	242 (98.4)	0.050, 1, 0.824
Posts depicting illicit drug consumption	731 (97.1)	493 (97.2)	238 (96.7)	0.021, 1, 0.885
Posts involving overt sexual content	715 (95)	481 (94.9)	234 (95.1)	0.000, 1, 1.000
Photos of a patient/client	705 (93.6)	493 (97.2)	212 (86.2)	32.119, 1, 0.000**
Swearing or foul language	658 (87.4)	439 (86.6)	219 (89.0)	0.685, 1, 0.408
Obscene gestures in photos (the middle finger, etc.)	647 (85.9)	433 (85.4)	214 (87.0)	0.226, 1, 0.634
Pictures of an individual clearly behaving drunkenly	632 (83.9)	424 (83.6)	208 (84.6)	0.047, 1, 0.828
Status updates describing substantial alcohol consumption at a party	615 (81.7)	418 (82.4)	197 (80.1)	0.471, 1, 0.493
Attitudes of superiority or condescending behaviour (assumed because of professional status)	608 (80.7)	411 (81.1)	197 (80.1)	0.050, 1, 0.824
Posts containing partial nudity	587 (78)	386 (76.1)	201 (81.7)	2.678, 1, 0.102
Endorsements of a pharmaceutical or health product without a conflict-of-interest disclosure	525 (69.7)	380 (75.0)	145 (58.9)	19.354, 1, 0.000**
Displaying membership in online groups dealing with controversial issues	405 (53.8)	280 (55.2)	125 (50.8)	1.127, 1, 0.288
Posts describing an interaction with a patient/client, while not revealing any identifying information	403 (53.5)	304 (60.0)	99 (40.2)	25.097, 1, 0.000**
Making opinionated comments about controversial issues	322 (42.8)	209 (41.2)	113 (45.9)	1.316, 1, 0.251
A picture of an individual having one alcoholic beverage	237 (31.5)	181 (35.7)	56 (22.8)	12.258, 1, 0.000**
Displaying your current relationship status	185 (24.6)	137 (27.0)	48 (19.5)	4.643, 1, 0.031*

*p < 0.05

**p < 0.01

PCA was conducted on 17 items in order to further clarify the underlying dimensions of the instrument. The Kaiser–Meyer–Olkin measure (0.855) and Bartlett’s test of sampling adequacy (χ^2^_136_ = 3652.305, *p* < 0.001) show that dimension reduction is applicable to these data. PCA extracted four components that cumulatively explained 54.41% of the total variance and can be theoretically explained. Varimax rotation was applied to the extracted components. The results of the PCA are shown in Table 5.

**Table 5:** Principal component analysis (PCA)

	Rotated component matrix	Component				Matrix recoding after FA
	Online behaviour	Somewhat unprofessional behaviour	Professional behaviour	Serious unprofessional behaviour	Illegal and condescending behaviour	
		1	2	3	4	
Somewhat unprofessional behaviour	Swearing or foul language	**0.792**	0.151	0.004	0.108	UPC
	Status updates describing substantial alcohol consumption at a party	**0.771**	0.224	0.034	0.029	UPC
	Obscene gestures in photos (the middle finger, etc.)	**0.761**	0.156	0.015	0.113	UPC
	Pictures of an individual clearly acting drunk	**0.761**	0.160	−0.003	0.043	UPC
	Posts involving overt sexual content	**0.592**	0.017	0.052	0.431	UPC
	Posts containing partial nudity	**0.535**	0.275	0.029	0.156	PC
Professional behaviour	Displaying membership in online groups dealing with controversial issues	0.260	**0.733**	−0.130	0.176	PC
	Making opinionated comments about controversial issues	0.155	**0.714**	−0.102	0.241	PC
	Displaying your current relationship status	0.185	**0.654**	0.116	−0.123	PC
	A picture of an individual having one alcoholic beverage	0.306	**0.605**	0.083	−0.209	PC
Serious unprofessional behaviour	Photos of a patient/client	−0.021	0.013	**0.782**	−0.038	UPC
	Posts disclosing information about a patient/client	0.091	−0.123	**0.660**	0.169	UPC
	Posts describing an interaction with a patient/client, while not revealing any identifying information	0.076	0.440	**0.454**	−0.039	PUPC
	Endorsements of a pharmaceutical or health product without a conflict-of-interest disclosure	−0.064	0.402	**0.419**	0.200	PUPC
Illegal and condescending behaviour	Petty criminal activity	0.341	−0.100	0.183	**0.631**	UPC
	Attitudes of superiority or condescending behaviour (assumed because of professional status)	0.002	0.444	0.000	**0.615**	UPC
	Posts depicting illicit drug consumption	0.484	−0.054	0.090	**0.517**	UPC

Loadings in bold indicate the highest component loading for each item (these being the most salient loadings for interpretation of each component)

*PC* professional content, *PUPC* potentially unprofessional content, *UPC* unprofessional content

The first component is called *somewhat unprofessional behaviour* and it is formed by items concerning content and posts that are unprofessional but do not invoke direct legal or formal consequences. Swearing, posts with alcohol and sexualized posts are part of this dimension. The second component is called *professional behaviour* and it encompasses content and posts on SNSs that are not unprofessional. The third component, called *serious unprofessional behaviour*, is formed by four items with extremely unprofessional and unethical contexts, some of which describe straight forward HIPAA violations. The last component is called *illegal and condescending behaviour* and it is formed by three items, two of which describe illegal activities, such as the consumption of illegal drugs and criminal actions, the last being expression of superiority on the basis of professional status.

PCA was used to obtain the final “professional” or “unprofessional” categorization of items of professionalism perceptions. With these two categories the ePACI index was used on the perception of e-professional behaviour by MDs and DMDs.

**Fig. 2:**
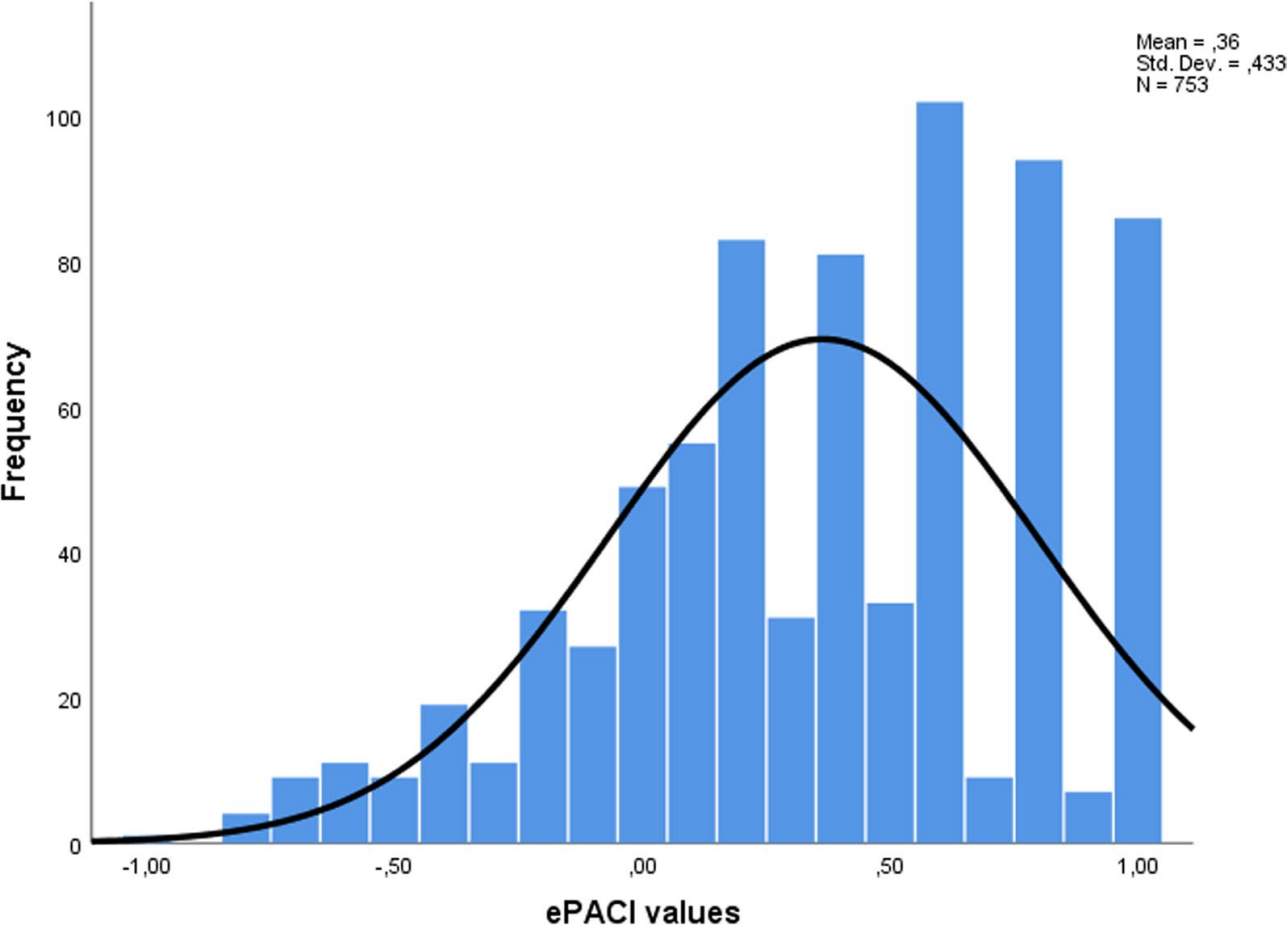
Histogram of distribution of ePACI values for MDs and DMDs

First, it was investigated whether average values of MDs and DMDs on the ePACI index were statistically significantly different from the correct answer. A t-test for independent samples showed that both MDs (t_506_ = 19.033, *p* < 0.001) and DMDs (t_245_ = 12.991, *p* < 0.001) statistically significantly deviated towards positive—conservatism from the “correct” neutral answer. Figure 2 represents a visual representation of the “conservative” deviation of ePACI values of both groups, with the mean of the value being 0.36 (SD = 0.433).

Second, differences between key groups on ePACI index values were investigated. There were no statistically significant differences in the ePACI index between MDs and DMDs (t_526.375_ = 1.206, *p* = 0.243), no differences between genders (t_327.019_ = 1.303, *p* = 0.193) nor those in public or private sector employment (t_745_ = 0.357, *p* = 0.722). There was a moderate positive correlation between age of the subjects divided into four age groups (< 35, 34–44, 45–54, 55+) and the index, with older HCPs more prone to deviate towards conservativism (r = 0.308, *p* < 0.001). When comparing the age groups, variance analysis was performed using the One-Way ANOVA test, which showed there was a statistically significant difference between the age groups in ePACI index values (F = 25.312, *p* < 0.001). A Tamhane T2 post hoc test for non-homogenous variance showed statistically significant differences between the < 35 and 45–54 age groups, with the < 35 age group having lower ePACI index values (*p* = 0.001), and the 55+ age group significantly higher ePACI index values (*p* < 0.001).

Also, there was a weak, but statistically significant correlation between the subjects’ access frequency and the index deviation towards the negative—liberal heading (r = −0.166, *p* < 0.001). As access frequency rose the subject was more prone to deviate towards a liberal e-professionalism assessment.

We tested the correlation between the number of the seven different SNS profiles we investigated (0–7) and the ePACI index, using the Spearman correlation coefficient, and we found a weak statistically significant negative correlation (r_s_ = −0.142, *p* < 0.001), meaning those who had more profiles on SNS had a more negative ePACI index value—a deviation towards liberalism. Deeper analysis of usage habits and SNS preferences was performed by exploring differences in the ePACI index regarding the number of SNSs used by respondents (Table 6). Users who had just one SNS profile had statistically significantly more positive—conservative values on the ePACI index than those with more than one. Also, users with just two profiles on different SNSs tended to have ePACI values more towards the positive than those users with more than two SNS profiles.

**Table 6:** Differences in the ePACI index between SNS sites used and varying numbers of SNSs available for use

	Mean	SD	df	t	*p*
Facebook					
Does not have a profile	0.49	0.493	64.317	2.105	0.039*
Has a profile^a^	0.35	0.426			
LinkedIn					
Does not have a profile	0.37	0.42	751	0.384	0.701
Has a profile	0.36	0.44			
Instagram					
Does not have a profile	0.41	0.449	751	2.370	0.018*
Has a profile	0.33	0.421			
SNS profile					
Has one profile	0.48	0.437	751	3.550	0.000**
Has a more than one profile	0.34	0.428			
Number of SNS profiles					
Has exclusively a Facebook profile	0.49	0.407	721	−3.267	0.001**
Has more than one SNS profile^b^	0.34	0.428			

*p < 0.05; **p < 0.01

^a^Can include more profiles on other SNSs

^b^Can include Facebook

There were no statistically significant differences between the MDs and DMDs regarding whether they would find it useful to have official guidelines for SNS usage. However, 25.9% of the subjects (n = 195) stated that they do not need additional guidelines.

## Discussion

SNSs have become ubiquitous in modern society. A large part of everyday information gathering, expression, communication, and social interaction has been transferred to this medium [49]. This phenomenon is also evident in our studied sample, as just over three quarters (75.4%, n = 753) of all surveyed and eligible respondents are SNS users. This is consistent with the findings of a recent scoping review, where investigated research reported from 50% to 80% of responders being SNS users [15]. Facebook is the predominant SNS (91.6%) while Instagram (63.1%) and LinkedIn (52.1%) are 2nd and 3rd respectively. Apart from the fact that these are the most important SNSs, the results mirror the distribution of SNS usage in the general population (GP) [50]. The median age of our studied population is 35 years, which places them directly at the nascent stage of SNSs and perhaps somewhat limits the knowledge and appeal for new and emerging SNSs like Snapchat (6%) and TikTok (4.6%). Twitter (10.4%) has never been a popular SNS in Europe or Croatia and YouTube is surprisingly low (32.9%), even though 71% of the Croatian GP uses YouTube [50]. This could perhaps be a “semantic” discrepancy because YouTube usage does not require an account, so not having a profile does not necessarily equate to a lack of use. However, there are interesting statistically significant differences between the two studied groups with DMDs being statistically significantly more likely to have an account on Facebook (χ^2^_1_ = 9.271, *p* < 0.01), Instagram (χ^2^_1_ = 19.350, *p* < 0.01), Snapchat (χ^2^_1_ = 4.967, *p* = 0.026) and TikTok (χ^2^_1_ = 8.862, *p* < 0.01). MDs are statistically significantly more present on LinkedIn (χ^2^_1_ = 6.637, *p* = 0.01) and YouTube (χ^2^_1_ = 6.584, *p* = 0.01). The differences can be partially explained by the fact that the DMDs in our studied population were predominantly (69.1%) and statistically significantly more (χ^2^_1_ = 164.481, *p* < 0.001) employed in private practice. It is possible that DMDs are using Facebook, Instagram and TikTok as marketing tools for self-promotion or advertisement of personal dental practices, as these are the main popular marketing platforms for advertising. Since the market in Croatia is saturated with DMDs and there is also a large dental tourism business, SNS platforms provide great tools and opportunities for advancing patient recruitment, patient communications and education [51]. One of the reasons for such differences in LinkedIn usage among the MD and DMD population in Croatia is that LinkedIn has been used recently as a platform for job recruitment of MDs, with a significant number of MDs emigrating from Croatia after being contacted by head-hunter agencies via LinkedIn or other SNS sites [52, 53]. YouTube is used as a teaching tool during undergraduate and postgraduate education for MDs, which could explain the difference in usage of that platform.

Regardless of individuals being active or passive in the online environment, all information, activities and actions on the internet are traceable once posted, so individuals have to act accordingly [31]. Even though many people are active on SNSs, only a smaller number are in careers that uphold certain professional standards, where the general public assumes they will behave professionally. In contrast to individuals from the general public, who can be passive in the virtual environment and are simply required to abide by general SNS rules for content, this small group of professions, which includes HCPs, according to the findings of a recent study should play an active role in online media [32]. By being required to follow, unwritten, stricter protocols, HCPs carry a greater burden of duties. This could make their self-perception of their e-professionalism much more rigorous. Results from our research reflect this in two ways. First, only 7.4% of the total HCPs in the sample state that they are “more active than passive” or “exclusively active”. This low activity on SNSs can be explained by a reluctance to post content that will be scrutinized more than content from sectors of the population that are not held to such a standard of professionalism. It is easier to play it safe by being inactive than to suffer the consequences of potential mistakes. Being inactive should not be confused with a lesser usage rate. Both groups are heavy users with 81.1% using SNS sites at least once a day, with DMDs being statistically significantly more online with access 5 to 10 or more times per day (U = 54,641.5, *p* = 0.003). Mobile devices are the preferred medium for accessing SNSs, with DMDs again being statistically significantly more inclined towards these devices than MDs (χ^2^_1_ = 11.258, *p* = 0.001).

To explain the second reflection on our research we must start by addressing the perception of professional behaviour. The perception of e-professional behaviour of our study group is very similar to that in previous research, both from our group involving the student population [13], as well as other authors’ research on practising HCPs [12]. Clear legal and HIPAA violations are perceived as highly unprofessional and there are no differences between groups in those items. However, the items “Photos of patients/client” and “Posts describing an interaction with a patient/client that does not reveal any identifying information” significantly differ with DMDs being more open to these items. Dental medicine is a field of medicine with a strong visual aesthetic, and educational content is most often conveyed through visual representations of treatment options. A recent study by Douglas et al. demonstrates that Instagram, being a very visually driven SNS, can be successfully used in dental education [54]. As DMDs are educated and consequently possibly desensitised to such content, they perhaps lose a high sensitivity of professionalism perception to such content. Finally, the item “Endorsements of a pharmaceutical or health product without a conflict-of-interest disclosure” also statistically significantly differs between MDs and DMDs. Pharmaceutical companies are not engaging aggressively with DMDs for drug/material/therapy application, as they do with MDs. This could explain DMDs not sensitized to such transgressions. As this instrument only examines individual items, overall perception could not be measured and we decided to use a coding scheme to categorize the items and develop the ePACI index.

As previous coding schemes had been developed for a different era of professionalism and especially in the wake of the “medbikini affair” [39, 55], a re-examination of professional standards became necessary and a SMePROF coding scheme that included a SMePROF rubric for the assessment of unprofessional content on Facebook was developed by Vukušić Rukavina et al. [40]. Categorizing of content has been arranged in three distinct groups: professional, potentially unprofessional, and unprofessional, taking into consideration new and updated perceptions of the professional online behaviour of HCPs. As the SMePROF coding scheme [40] can now be percieved as the centre value for e-professionalism, tendencies of professionalism can be explored: either towards a more lenient, relaxed perception—liberal, or a more cautious and rigid perception—conservative. PCA confirmed our initial coding of the modified White et al.’s e-professional behaviour perception items [12]. Four PCA components can be categorized into two fields: professional (*somewhat unprofessional behaviour, professional behaviour*) and unprofessional (*serious unprofessional behaviour*, *illegal and condescending behaviour*).

Behaviours in items forming the component *somewhat unprofessional behaviour* could be unwanted because they shed a bad light on the perception of HCPs on SNS [14]. The component *professional behaviour* experienced the most revisions from its perception in previous literature and introduced context as an important factor in judging a post on SNS. Showing alcohol consumption is unprofessional only in certain and very specific contexts (such as in the medical/dental office, or while wearing physician’s attire) [40]. Also, the opportunity to express opinions on SNSs is something that all users have and should not be perceived as damaging to professionalism. Excluding MDs and DMDs diminishes their basic right of free speech and robs the overall discussion of another (HCP’s) opinion [56].

Behaviour and posts in the *serious unprofessional behaviour* remained in the realm of activity which should evoke a formal response from governing bodies [15, 29].

Finally, the component of *illegal and condescending behaviour* is formed by two items describing illegal activities, and interestingly a third item—expressing superiority based on professional status. Several studies, including our current results, have reported the first two items as being the highest ranked in the perception of unprofessional behaviour [12, 13, 57]. The fact that the item expressing superiority based on professional status aligned with the last component (*illegal and condescending behaviour*) instead of with, for example, the first (*somewhat unprofessional behaviour*) shows that DMs and DMDs are very conscious of this kind of behaviour, its ethical and social consequences and the potential for poor reputation that it can represent.

While reviewing literature, although there are available scales and indexes in some form or another for the student population [44, 58], we did not encounter a way to easily measure and scale perceptions of e-professionalism among graduate MDs and DMDs.

The ePACI index provides a simple tool that allows us to assess and compare professionalism perceptions of and between different groups of HCPs. The deviation towards liberal may expose HCPs more to the dangers of SNSs and those HCPs need to be educated about the dangers of SNSs, or there could be negative impacts to their professional reputation. Those respondents who fall into the conservative category may be more sensitive to the dangers of SNSs. It could also be understood as some form of self-protection mechanism that shields users from the dangers, but may also limit their ability to exploit all the benefits SNSs provide. This is important for all HCPs because these deviations can have serious, although contrasting, effects on e-professionalism.

In this study the ePACI index values deviated for both MDs and DMDs towards the positive—conservative (mean = 0.36, SD = 0.433), with no statistically significant differences between the two groups. This overall coherence of perception, and a deviation to a more careful consideration of professional behaviour on SNSs can be explained by the higher expectation of HCPs themselves regarding their online presence, and their awareness of the general public’s assumption that they will behave professionally. This hypothesis integrates well with our results of HCPs’ unwillingness to act actively on SNSs, but rather be more passive, thus shielding themselves from peer and public scrutiny. Interestingly, even employment type did not a have a significant influence on the ePACI values of both groups, perhaps emphasizing more the underlying high standards of professionalism perception even in the face of business-driven motives. The age of the respondents, however, did have a statistically significant influence on ePACI values. Our results show that the increasing age of HCPs correlated with a deviation slightly towards the positive values of the ePACI index. Also, there are statistically significant differences between respective age groups, with the digital natives (< 35 yrs. old) being the most liberal of the four examined age groups. Possible explanations for this are that younger HCPs are more versed in how to navigate SNSs and are more accustomed to SNSs being a part of their identity [2]. This can lead them to be more error-prone and more likely to post unprofessional content [12, 14, 59]*.* This was also seen in the definitive results of the mixed methods study conducted by Vukušić Rukavina et al., which found more unprofessional content on the Facebook profiles of students (5.8%) than faculty (0%) [40]. Age can also play a role in the terms “older and wiser”, meaning more cautious about posting unprofessional behaviour online, was proven in studies comparing students’ and faculties’ online behaviour [15]. Medical students were proven more likely than faculty to display content they would not want patients to see (57% vs. 27%), report seeing inappropriate content on colleagues’ SNS profiles (64% vs. 42%), and ignore harmful postings by colleagues (25% vs. 7%)” [47]. Another predictor of liberal perceptions is the access frequency: the higher the daily consumption of SNSs, the more liberal the perception. This is again in line with the idea of comfort and knowledge of SNS usage and higher digital literacy skills. The final piece of evidence adding to this idea is the number and type of SNS profiles correlation to ePACI. Not having one of the seven major SNS profiles we included in our questions, or having only a Facebook profile (today's “old person’s SNS”) was a statistically significant predictor of having a more conservative perception of e-professionalism. The more SNS profiles one had (between 2 and 7 profiles), and the more “modern” they were (Instagram, LinkedIn), the more values deviated towards liberal. We perceive any deviation (either towards liberal or conservative) from the centre value as an error in perception. However, this mechanism of perceiving all such behaviour as unprofessional brings with it the danger of missing the opportunities that SNSs bring to medical care, professional reputation, business, and so on [15]. This emphasizes the need for education—not only for nascent HCPs during graduate studies but for all levels of HCPs, particularly those in the latter stages of their careers. Walton et al. have stressed that there is a need for instructions for use of these developing media and communication training for HCPs before they begin using SNSs for their professional work [60]. Mosalenajad et al. indicate that education can improve professionalism on SNSs and set appropriate boundaries between HCPs and the public, but also how it can help HCPs embrace and execute the active role society expects from them as professionals [32]. Recognizing one's need for further education is a step in the right direction, and 74.1% of our respondents stated that they would benefit from additional guidelines on e-professionalism. Exposure to problems of e-professionalism by opening debate and dialogue with HCPs at all levels to raise attention and awareness, especially on conflicting or blurred issues, could perhaps alert an even larger percentage of HCPs to the need for guidelines on e-professionalism [15, 32, 59]. The best example of a successful debate is the #medbikini movement, which sparked awareness, opened a debate, and led to the re-examining and directing of e-professionalism towards a new, better understanding of professionalism on SNSs [40].

## Strengths

This paper represents the first-time e-professionalism perception has been able to be measured and compared. The ePACI index is a novel tool for an evaluation of deviation in perception, which can give information and guidance on where efforts in oversight, regulation and education should be steered. This paper provides the instruments necessary for other researchers to create an ePACI index, which can be adjusted depending on the instruments used. In order to make construction of an index more transparent and easier to replicate by other researchers, a general formula for index standardization is provided in the paper. In the event that the number of items for perception of e-professional content on SNSs in a future instrument were changed (for example if the targeted population were a student population and all original items were used), the index can still be standardized to a range from −1 to +1 using the general formula. Also, it allows for comparisons of different HCP population groups and analysis of their specific perceptions of e-professionalism. In this paper results from a questionnaire that was sent out to the complete mailing lists of members of the CMC and the CDMC are shown. A high number of responses (1013 responses) was obtained, with the results having a sufficient number of respondents for the required statistical tests (including the multivariate test [45]). This paper also shows—for the first-time—demographics of a convenience non-probabilistic sample used in this study, with 753 respondents, MDs and DMDs in Croatia who are SNS users enabling us to present their habits and perceptions of e-professionalism. This will serve as a basis for the creation of guidelines that will be adopted as official documents of the CMC and the CDMC. Our results demonstrate differences in attitudes between MDs and DMDs that should be considered when creating the guidelines.

## Limitations

Participation was voluntary and the sample selection was limited. Our study is susceptible to information and recall bias, as it was based on a non-probabilistic convenience sample with self-reporting by the MDs and DMDs. The low response rate is acknowledged; however, it is to be expected from an mailing-lists survey [61].

Achieving a high response rate for physicians has been challenging, and with response rates declining in recent years, innovative methods are needed to increase rates [62].

Both of these populations, because of their high social status, busy hours and fragmented workplaces can be considered hard-to-reach populations [63], especially for cross-sectional survey. E-mail survey, sent through respective chambers newsletter, inviting them to participate in this study’s survey, was the only way to approach that population. Unfortunately, overall physician response rates are declining [64–68], and web surveys have overall lower response rates than other survey methods [69], the combination that could explain lower response rate in this research too.

There is a possibility of non-response bias in this study. In non-probability surveys the bias has the potential to be much greater, since it is likely that those who opt-in are not representative of the general population. Furthermore, in non-probability surveys there is often no way to assess the potential magnitude of the bias, since there is generally no information on those who chose *not* to opt-in [70].

Comparison between the ratios of MDs and DMDs that were surveyed (67.1% MDs vs. 32.9% DMDs) and respondents (70.1% MDs vs. 29.8% DMDs) with respondents SNS users (67.3% MDs vs. 32.7% MDs) shows very close consistency in the proportionality. But we cannot conclude that 4.4% respondents were representative of the entire population that was surveyed.

It wasn’t possible to compare demographic data of our respondents with the entire population that was surveyed because CMC and CCDM couldn’t provide us with demographic variables of the population of the MDs/DMDs (sex, age, type of employment: private or public, etc.) due to the protection of their data and GDPR regulations [71, 72]. Thus, we can only speculate about the reasons of low RR and potential non-response bias. Possible reasons could be: newsletter invitations sent by respective chambers were not seen by study population (e-mail went to the spam or promotions section), not being a SNS user might affect willingness to participate in the study, the study questionnaire had 40 questions, some of the respondents could have started to answer, but due to longer time then expected to finish it, they decided not to finish it.

The chambers do not have information about whether their members are SNS users or not, therefore it was not possible to send invitations to participate in this research only to those who are SNS users. Instead, we used a selection question and eliminated those who do not use SNS. But this also means that we were forced to include in our sampling frame all members of the respective chambers. If we could have sent participation link only to the population of interest (SNS users), our sampling frame would be different and response rate would be higher.

Even though we can’t control possible non-response bias, mitigating circumstances are that prior research suggests that physician surveys may be less prone to non-response bias than surveys of other populations, given that physicians are rather homogenous with respect to their knowledge, attitudes, and beliefs [73].

As the ePACI index is based upon the evaluation of e-professionalism perception by HCPs and not from the perception of the general population, a skewing of the base value (correct assessment—0) could have occurred. This is the next goal of the SMePROF project: to evaluate the perception of the general population regarding HCP’s professionalism on SNSs. The current study is based on Croatian MDs and DMDs which limits the generalizability of the results regarding other populations, and other HCPs (in nursing, pharmacy, nutrition, and so on).

Even though a convenience non-probabilistic sample does not allow inference to the population, it allows us to see indicative tendencies that could provide some insights into e-professionalism and to perform multivariate analyses needed for third and fourth study aims (to develop an e-professionalism assessment compatibility index and to investigate tendencies and differences among MDs and DMDs in Croatia of MDs and DMDs in Croatia). Those results and differences are not inferred to population, but are used as indications and new pieces of information about hard-to-reach population that could guide other interested scientists’ future research.

However, as the ePACI index provides numerical results, comparisons with future research using the ePACI index could provide insight into the similarities and differences among geographically different populations of HCPs.

## Conclusions

Exploration of SNS influence within the HCP community in Croatia proved worthy of interest, with all four aims successfully attained. Both MDs and DMDs in Croatia are frequent SNS users, with Facebook and Instagram being the main SNS sites used. Both groups have a good understanding of e-professionalism; however, DMDs are less inclined to view visual depictions of patients on SNSs and endorsements from pharmaceutical companies without conflict-of-interest disclosure as unprofessional. The newly developed ePACI index deviates slightly towards the conservative side for both groups, with HCPs who are older, who have fewer SNS profiles, and who access their profiles less frequently and are therefore not using SNS a lot having more statistically significantly more conservative ePACI index values. The development of the ePACI index, and its subsequent usage in further research, will have a direct influence in gathering comparable data and will be able to direct efforts in oversight, regulation and education.

## Supplementary Information

**Additional file 1.** SMePROF Project Survey Questionnaire on Social Media Usage, Attitudes, Ethical Values and E-professional Behaviour of Doctors of Medicine and Doctors of Dental Medicine PDF file containing full questionnaire used in this research.

## Data Availability

The datasets used and/or analysed during the current study are available from the corresponding author on reasonable request.

## Abbreviations

CCDM: Croatian Chamber of Dental Medicine
CMC: Croatian Medical Chamber
DMD: Doctor of dental medicine
ePACI: E-Professionalism Assessment Compatibility Index
GP: General population
HCP: Healthcare professional
HIPAA: The Health Insurance Portability and Accountability Act
MD: Medical doctor
PCA: Principal component analysis
SM: Social media
SMePROF: Social Media e-Professionalism
SNS: Social networking site
