# Supplementary material for: Differences between doctors of medicine and dental medicine in the perception of professionalism on social networking sites: the development of the e-professionalism assessment compatibility index (ePACI)
Source: BMC Med Ethics. 2022 Dec 6;23:129. doi: 10.1186/s12910-022-00870-0 (PMC9727956; doi:10.1186/s12910-022-00870-0)
Supplement: Supplementary file 1 — Additional file 1. SMePROF Project Survey Questionnaire on Social Media Usage, Attitudes, Ethical Values and E-professional Behaviour of Doctors of Medicine and Doctors of Dental Medicine PDF file containing full questionnaire used in this research. [file 12910_2022_870_MOESM1_ESM.pdf]

1. I hereby confirm that I have been informed of the objectives of this research and agree to participate in it.

1. I agree
2. I do not agree

1. continues to the next question, 2. ends the survey.

2. What gender are you?

1. male
2. female

3. How old are you?

______

4. Your workplace is[1]:

1. health institution within a public sector
2. private health institution
3. university/faculty position only
4. combination of private and public institution
5. combination of faculty position and health institution
6. other (please enter)

6. continues to the next question, other jump to question 6.

5. Please enter the type of your workplace. ________

6. Please select your status according to specialization:

1. doctor of medicine, not in a residency program
2. doctor of medicine, resident
3. doctor of medicine, specialist
4. dentist, not in a residency program
5. doctor of dental medicine, resident
6. doctor of dental medicine, specialist

6.2 and 6.3. continue to the next question. 6.5 and 6.6 skip to question 8. 6.1 and 6.4 skip to question 9.

[1] This is closest possible translation from Croatian language. The typology of jobs is adapted for measurement in the Croatian health-care system and Croatian context. This question should be adapted if it would be used in another country.

7. and 8. Select the completed residency / residency in progress:

7. (5.2, 5.3) For doctors of medicine:	8. (5.5, 5.6) For doctors of dental medicine:
1. Abdominal surgery	1. Paediatric dentistry
2. Allergology and clinical immunology	2. Endodontics with restorative dentistry
3. Anaesthesiology, resuscitation and intensive care	3. Family dentistry
4. Dermatology and venereology	4. Oral surgery
5. Child and adolescent psychiatry	5. Oral medicine
6. Paediatric surgery	6. Orthodontics
7. Endocrinology and diabetology	7. Periodontology
8. Epidemiology	8. Dental prosthetics
9. Physical medicine and rehabilitation	
10. Gastroenterology	
11. Geriatrics	
12. Gynaecology and obstetrics	
13. Haematology	
14. Emergency medicine	
15. Infectious diseases	
16. Internal medicine oncology	
17. Public health medicine	
18. Cardiology	
19. Cardiothoracic surgery	
20. Clinical pharmacology with toxicology	
21. Clinical microbiology	
22. Clinical radiology	
23. Laboratory immunology	
24. Laboratory medicine	
25. Maxillofacial surgery	
26. Occupational and sports medicine	
27. Nephrology	
28. Neurosurgery	
29. Neurology	
30. Nuclear medicine	
31. Family medicine	
32. Ophthalmology and optometry	
33. Oncology and radiotherapy	
34. General internal medicine	
35. General surgery	
36. Orthopaedics and traumatology	
37. Otorhinolaryngology	
38. Pathology and cytology	
39. Paediatrics	
40. Paediatric Infectious Diseases	
41. Plastic, reconstructive and aesthetic surgery	
42. Psychiatry	
43. Pulmonology	
44. Rheumatology	
45. Forensic medicine	
46. School and adolescent medicine	
47. Transfusion medicine	
48. Urology	

49. Vascular surgery	

9. How often do you treat family members or close friends?

1. Never
2. Rarely
3. Occasionally
4. Often

10. Do you use a social networking site? (e.g., Facebook, Instagram, LinkedIn, etc.)?

1. Yes
2. No

If "2. No "then continue to the next question. If "1. Yes, then continue to question 12.

11. To what extent is each of the above reasons why you do NOT use social networking sites?

	Not at all	Yes, to a lesser extent	Yes, to a greater extent
1. Lack of knowledge	1	2	3
2. Lack of time	1	2	3
3. Lack of interest	1	2	3
4. Lack of perceived value	1	2	3
5. Concern about harm to professional image	1	2	3

Complete a survey for those who do not use networks.

12. Your profile on social networking site is:

	One profile under my real name	One profile, but with a different name (pseudonym)	Profile with real and different name (pseudonym)	I have more than one profile, private and professional	I don't have a profile
1. Facebook	1	2	3	4	99[2]
2. Twitter	1	2	3	4	99
3. LinkedIn	1	2	3	4	99
4. YouTube	1	2	3	4	99
5. Instagram	1	2	3	4	99
6. TikTok	1	2	3	4	99
7. Snapchat	1	2	3	4	99

13. How skilled are you in using the following social networking sites?

[2] Code '99' is used for missing values that are not 'system missing values' (for example skipped question). The number 99 is used to make it easier to spot potential data processing errors.

	Not at all	Beginner	Somewhat skilled	Extremely skilled
1. Facebook	1	2	3	4
2. Twitter	1	2	3	4
3. LinkedIn	1	2	3	4
4. YouTube	1	2	3	4
5. Instagram	1	2	3	4
6. TikTok	1	2	3	4
7. Snapchat	1	2	3	4

14. How selective are you when accepting friend / follow requests on your PRIVATE profile of the following social networking sites?

	I accept requests ONLY from friends I know well and members of my family	I accept requests from people I have met in person	I accept requests from both famous people and those I have never met in person but my name sounds familiar	I accept all requests regardless of whether I know them personally or not	I don't use that social networking site
1. Facebook	1	2	3	4	99[3]
2. LinkedIn	1	2	3	4	99
3. Instagram	1	2	3	4	99
4. TikTok	1	2	3	4	99
5. Snapchat	1	2	3	4	99

15. To what extent is each of the below reasons why you use social networking sites?

	Not at all	Yes, to a lesser extent	Yes, to a greater extent
1. To stay in touch with current friends and family.	1	2	3
2. To connect with old friends I lost contact with.	1	2	3
3. Professional communication (networking and education).	1	2	3

[3] Code '99' is used for missing values that are not 'system missing values' (for example skipped question). The number 99 is used to make it easier to spot potential data processing errors.

16. To what extent do you agree with the following statements on social networking sites?

	I don't agree at all	I disagree	I don't know, I'm not sure	I agree	I totally agree
1. Potential and/or existing employers may use information found on social networking sites to make decisions about prospective and/or existing employees.	1	2	3	4	5
2. Social networking sites are just a fad.	1	2	3	4	5
3. Social network sites are a waste of time.	1	2	3	4	5
4. The emergence of social networking sites illustrates a growing need among people for a sense of community.	1	2	3	4	5
5. Social networking sites allow people with similar interests to stay connected.	1	2	3	4	5
6. Social networking sites are a great way for people to stay in touch with one another.	1	2	3	4	5
7. It consumes too much time to maintain and/or read social networking site.	1	2	3	4	5
8. Social networking sites could be an effective means of communication for my health institution / practice.	1	2	3	4	5
9. Social network sites are a great way to build online communities of people with shared interests or traits.	1	2	3	4	5
10. Social networking sites have great potential for marketing businesses and/or individuals.	1	2	3	4	5
11. I want to read about my friends and/or family members on their social network sites.	1	2	3	4	5
12. I do not care what other people are doing on social networking sites.	1	2	3	4	5
13. It is important that the healthcare professional has their own profile on social networking sites where they can present themselves and their activities.	1	2	3	4	5

17. How often do you track your online presence (e.g., “google” your name, search for your images on g-image, etc.)?

1. Never
2. Once a year or less
3. About once a month
4. About once a week
5. Almost every day
6. Several times a day

1. jumps to question 19. The others continue to the next question.

18. To what extent is each of the below the reason you monitor your online presence?

	Not at all	Yes, to a lesser extent	Yes, to a greater extent
1. In order to make sure that the published information is true.	1	2	3
2. In order to make sure that the published information is complete	1	2	3
3. To make sure that the published information is professional.	1	2	3

19. Have you ever discovered that information about you on social networking sites is:

	Yes	No
1. Incorrect	1	2
2. Incomplete	1	2
3. Unprofessional	1	2

20. Has anyone else ever posted material about you on a social networking site that you found embarrassing or where you acted unprofessional?

1. Yes
2. No

1 continues to the next. 2 jumps to 22.

21. What did you do if you found such information?

	Yes	No
1. I deleted people from my friends list	1	2
2. I deleted comments from my profile	1	2
3. I untagged myself.	1	2

22. What do you usually use social networking sites for?

1. solely for personal purposes
2. mostly for personal purposes, less for professional
3. for both personal and professional purposes
4. mostly for professionals, less for personal
5. for professional purposes only

23. To what extent do each of the below worry you when using social networking sites?

	Does not worry me at all	It worries me somewhat	It worries me a lot
1. Public perception of unprofessional behaviour on my part.	1	2	3
2. My family's perception of unprofessional behaviour on my part.	1	2	3
3. Public perception of the unprofessional behaviour of my colleagues.	1	2	3
4. Public perception of my profession.	1	2	3
5. Violation of patient confidentiality.	1	2	3
6. Publishing inaccurate medical / dental information available to patients.	1	2	3

24. How often do you check your social networking site profiles?

1. More than 10 times a day
2. 5-10 times a day
3. 2-4 times a day
4. Once a day
5. 2-3 times a week or less often

25. From which device do you most often access social networking sites?

1. Mobile / tablet
2. Personal computer (desktop or laptop)

26. Have you ever changed the basic privacy settings on the social networking site you use the most?

1. Yes
2. No
3. I don't know

If 1 continue to the next question. If 2 or 3 jumps to question 28.

27. Please indicate all the reasons why you changed your privacy settings on social networking site:

	Yes	No
1. Protection of my personal data from unknown persons	1	2
2. The risk that my profile could be seen by a current or future employer	1	2
3. I held a position for which I had to be a role model to others (mentor, teacher, demonstrator, etc.)	1	2
4. Advice from colleagues and friends	1	2
5. I didn't believe in the security of the initial social networking site privacy settings	1	2

28. What are the privacy settings on your PRIVATE profile on the following social networking sites:

	Completely public (anyone can see the complete content of your profile)	Limited public visibility (strangers / people outside your "friends" circle may see some information, but not the entire content of your profile)	Completely private (only people you have accepted as "friends" can see your profile)	I do not know	I don't use that social networking site
1. Facebook	1	2	3	4	99[4]
2. Instagram	1	2	3	4	99

29. How would you describe your usual actions or behaviour on social networking site with respect to the following definitions?

Active behaviour: commenting, posting links, or photo sending invitations

Passive behaviour: reading, viewing, viewing your own photos or other content that pertains to you but has been posted by others

1. Exclusively passive
2. More passive than active
3. Equally active and passive
4. More active than passive
5. Exclusively active

30. Have you ever posted or commented on something on social networking site and later regretted it?

1. Yes
2. No

31. Have you ever visited a profile of a patient or a member of a patient's family on social networking ite?

1. Yes, patient profile
2. Yes, family member profile
3. Yes, both
4. No.

If (1, 2, 3) proceed to the next question. If 4 jump to question 33.

32. What is the reason for visiting the profile of the patient or a member of the patient's family?

	Yes	No

[4] Code '99' is used for missing values that are not 'system missing values' (for example skipped question). The number 99 is used to make it easier to spot potential data processing errors.

1. Obtaining more information for the purpose of patient care	1	2
2. Social communication	1	2
3. Out of curiosity	1	2

33. Has a patient or family member ever sent you a request to your private profile on a social networking site?

1. Yes, patient
2. Yes, a member of the patient's family
3. Yes, both
4. No.

If (1, 2, 3) proceed to the next question. If 4 jump to question 35.

34. Do you accept requests for "friendship" from patients or their family members??

	Never	Rarely	Sometimes	Always
1. From the patient	1	2	3	4
2. From a member of the patient's family	1	2	3	4

35. Have you ever sent a "friend request" from a private profile on a social networking site to a patient or a member of the patient's family?

1. Yes, to the patient
2. Yes, a family member
3. Yes, both
4. No.

36. How often have you personally acted in following ways on ANY SOCIAL NETWORKING SITE THAT YOU USE and on which you have a private profile? If you've never been in a situation where this behaviour could have happened, select "I have never been in a situation where this can happen".

	Never	Rarely	Occasionally	Often	I have never been in a situation where this can happen
1. I asked for permission from a colleague to mention him in the post.	1	2	3	4	99[5]
2. I shared some information about the patient that I received via social networking site with other people.	1	2	3	4	99
3. I posted a photo of my patient without his/her knowledge.	1	2	3	4	99

[5] Code '99' is used for missing values that are not 'system missing values' (for example skipped question). The number 99 is used to make it easier to spot potential data processing errors.

4. I have included patient information that I learned through social networking sites in medical records without his/her knowledge.	1	2	3	4	99
5. I shared medical / dental advice on social networking sites without my name being visible.	1	2	3	4	99
6. By posting on social networking sites, I reveal medical / dental myths and misinformation.	1	2	3	4	99
7. Depending on the appropriateness of the content of my posts, I determine to whom which post will be visible.	1	2	3	4	99
8. I share posts on social networking sites that contain general medical / dental advice.	1	2	3	4	99
9. If I notice that someone else has posted something about me (e.g. my picture, location or similar), I control to whom it will be visible.	1	2	3	4	99
10. I have published content that depicts informal situations in my workplace (e.g. drinking with colleagues, parties at work, etc.).	1	2	3	4	99
11. I use social networking sites to raise public awareness about public health actions.	1	2	3	4	99
12. I have published some information about my patient.	1	2	3	4	99
13. From a private profile, I communicate with patients regarding medical / dental problems and treatment.	1	2	3	4	99
14. On social networking site, I choose which patients I will contact and which I will not.	1	2	3	4	99
15. I use my profile to share information about new scientific knowledge in the field of medicine / dental medicine.	1	2	3	4	99
16. I post on social networking site calling for responsible health behaviour.	1	2	3	4	99
17. In the posts, I make sure that my expression is completely professional.	1	2	3	4	99
18. In the posts, I clearly differ my personal opinion about a medical / dental issue from scientifically based facts.	1	2	3	4	99
19. I use swear words or some other vulgar expression in my posts.	1	2	3	4	99

37. To what extent do you agree with the following statements regarding the use of social networking sites by doctors / dentists?

	I don't agree at all	I disagree	I don't know, I'm not sure	I agree	I totally agree
1. As a doctor of medicine / dental medicine, it is my duty to follow current trends in the use of social networking sites.	1	2	3	4	5

2. Guiding patients on social networking sites is a new duty of doctors / dentists in the digital age.	1	2	3	4	5
3. It is not always possible to fully maintain professionalism in online activities.	1	2	3	4	5
4. Social networking sites have jeopardized the balance of private and professional lives of doctors / dentists.	1	2	3	4	5
5. Due to the constant visibility on social networking sites, professionals are not able to fully relax.	1	2	3	4	5
6. Due to the excessive risk, the use of social networking sites should be RESTRICTED to health professionals.	1	2	3	4	5
7. Due to the high risk, health professionals should be BANNED from using social networking sites.	1	2	3	4	5
8. I don't think my online activities affect me as a professional.	1	2	3	4	5
9. I need to be able to do whatever I want online.	1	2	3	4	5
10. My employer has no right to interfere in my online activities.	1	2	3	4	5
11. It is ethically acceptable for a doctor / dentist to visit a patient's profile on social networking sites.	1	2	3	4	5
12. It is ethically acceptable for a doctor / dentist to communicate with a patient through social networking sites as part of patient care and health care.	1	2	3	4	5
13. It is ethically acceptable for a doctor / dentist to communicate with a patient through personal social networking sites without it being part of the patient's health care.	1	2	3	4	5
14. Social networking sites have the potential to improve communication between doctors / dentists and patients.	1	2	3	4	5
15. Communication with the patient through social networking sites can be achieved without compromising the confidentiality of the doctor / dentist-patient relationship.	1	2	3	4	5
16. It is inadmissible to share privileged information about the patient on social networking sites without their consent.	1	2	3	4	5

38. Consider the following list of possible negative outcomes resulting from unprofessional online behaviour and indicate the extent to which you agree.

	I don't agree at all	I disagree	I don't know, I'm not sure	I agree	I totally agree
1. People may misjudge you solely on the basis of published content.	1	2	3	4	5
2. There is a possibility that your online behaviour may affect the perception of others in your profession.	1	2	3	4	5

3. It is possible that a potential employer will not hire you or invite you for a job interview due to information revealed about you online.	1	2	3	4	5
4. It is possible to lose a position you already have (as an employee or trainee) due to information revealed about you online.	1	2	3	4	5

39. Which of the following types of posts, published on PRIVATE social networking sites of doctors / dentists, do you consider unprofessional?

	Yes	No
1. A picture of an individual having one alcoholic beverage	1	2
2. Pictures of an individual clearly acting drunk	1	2
3. Status updates describing substantial alcohol consumption at a party	1	2
4. Posts depicting illicit drug consumption	1	2
5. Posts that disclose information about a patient/client	1	2
6. Photos of a patient/client	1	2
7. Posts describing an interaction with a patient/client, that do not reveal any identifying information	1	2
8. Swearing or foul language	1	2
9. Obscene gestures in photos (the middle finger, etc.)	1	2
10. Petty criminal activity	1	2
11. Endorsements of a pharmaceutical or health product without a conflict-of-interest disclosure	1	2
12. Posts involving overt sexual content	1	2
13. Posts that contain partial nudity	1	2
14. Displaying your current relationship status	1	2
15. Displaying membership in online groups dealing with controversial issues	1	2
16. Making opinionated comments about controversial issues	1	2
17. Attitudes of superiority or condescending behaviour (assumed because of professional status)	1	2

40. Do you think it would be useful for you to have official guidelines on how to use social networking sites professionally?

1. Yes
2. No
